# Breastfeeding Prevalence in Austria according to the WHO IYCF Indicators—The SUKIE-Study

**DOI:** 10.3390/nu13062096

**Published:** 2021-06-19

**Authors:** Bernadette Bürger, Karin Schindler, Tanja Tripolt, Hans Peter Stüger, Karl-Heinz Wagner, Adelheid Weber, Alexandra Wolf-Spitzer

**Affiliations:** 1 Division Integrative Risk Assessment, Data and Statistics, Austrian Agency for Health and Food Safety (AGES), Spargelfeldstraße 191, 1220 Vienna, Austria; tanja.tripolt@ages.at (T.T.); hans-peter.stueger@ages.at (H.P.S.); alexandra.wolf-spitzer@ages.at (A.W.-S.); 2 Department of Nutritional Sciences, University of Vienna, Althanstraße 14, 1090 Vienna, Austria; karl-heinz.wagner@univie.ac.at; 3 Federal Ministry of Social Affairs, Health, Care and Consumer Protection, Stubenring 1, 1010 Vienna, Austria; karin.schindler@meduniwien.ac.at (K.S.); adelheid.weber@gesundheitsministerium.gv.at (A.W.); 4 Division of Endocrinology and Metabolism, Department of Internal Medicine III, Medical University of Vienna, Währinger Gürtel 18-20, 1090 Vienna, Austria

**Keywords:** breastfeeding, initial breastfeeding, exclusive breastfeeding, breastfeeding prevalence, breastfeeding duration, IYCF indicators, longitudinal, monitoring, infant formula

## Abstract

Breastfeeding and infant nutrition have an important impact on child health. The last representative data on breastfeeding in Austria was collected in 2006. The SUKIE-Study (Säuglings- und Kinderernährung) is a representative, longitudinal survey (online questionnaire) for participating mothers at four time points (14 days, four, six and 12 months post-partum). Questions on when other foods were first introduced were asked retrospectively. To ensure international comparisons, the World Health Organization’s definitions for breastfeeding, including “Infant and Young Child Feeding” indicators, were used. After eligibility screening, 1214 of 1666 invited mothers were included in the analysis. The initial breastfeeding rate was 97.5% and was reduced to 40.8% after 12 months. The rate of exclusive breastfeeding at one week of age was 55.5% and decreased to 1.9% after six months. Half of the infants received infant formula for the first time within the first three days of life (median). Out of the mothers that did wean breastfeeding in the first 12 months, the median duration was 27 weeks (right-censored data). Compared with 2006, an increase (93.2% to 97.5%) in the initial breastfeeding rate was found. However, other findings show that breastfeeding duration, including exclusive breastfeeding rates, need further improvement.

## 1. Introduction

Breastfeeding is an important contributor to child health. It immunizes the infant for the first time, may improve IQ (intelligence quotient), provides protection against respiratory infections and certain non-communicable diseases, and redounds as a primary prevention of obesity, among others [1,2,3,4,5,6]. Therefore, it is crucial to promote the importance of optimal nutrition as early as possible. One of the global targets for nutrition is to increase the rates of exclusive breastfeeding (EBF) in the first 6 months from 41% to 70% by 2030 [7]. The World Health Organization (WHO) reported that between 2006 and 2012 in the WHO European Region, only approximately 25% of infants were exclusively breastfed within the first six months [8,9]. Shorter durations of breastfeeding are more common in high-income countries [10].

Monitoring of breastfeeding prevalence and breastfeeding behavior, as well as infant and child nutrition, is fundamental for deriving appropriate promotion measures and is therefore an essential part of national and international strategies and recommendations in the field of child health [4,11,12,13,14]. Regular monitoring and up-to-date information on breastfeeding trends [15] are also recommended to achieve the global nutrition targets including “there is no increase in the rate of overweight in children under five years of age” [4].

Promotion and support of breastfeeding is essential for achieving most of the Sustainable Development Goals (SDG) [10] such as target 2.2, where it is committed that all forms of malnutrition, including overweight and obesity, should be stopped by 2030 [2,16].

The most recent representative study on breastfeeding behavior and infant nutrition in Austria was published by the Federal Ministry of Health in 2006 [17]. The data at that time showed that 93% mothers initiated breastfeeding. After 6 and 12 months, only 55% and 17%, respectively, were breastfeeding. At the age of five months, more than 70% of the infants had received formula for the first time [17]. Compared with data from other European countries (from 1998 to 2013), Austria had one of the highest rates for breastfeeding within an hour of birth. With regard to exclusive breastfeeding under four months and under six months of age, Austria was midrange. One year after birth, the breastfeeding rate in Austria was lower compared with other European countries (median 28%) [18]. A closer look at breastfeeding rates in other German-speaking countries shows that the initial breastfeeding rates were similar in Germany and Switzerland, with 96.6% and 95%, respectively [19,20]. However, due to the lack of standardized survey methods for the monitoring of breastfeeding, as well as the inconsistent use of definitions (such as including water for exclusive breastfeeding), data comparability between different countries is limited [18,21]. 

WHO and UNICEF (United Nations International Children’s Emergency Fund) recommend that data on exclusive breastfeeding (EBF) is collected every five years and to complete an assessment from the World Breastfeeding Trend Initiative (WBTi) to reach the target of 75% of countries reporting data regularly on breastfeeding prevalence by 2030 [7].

Since data on breastfeeding behavior have not been updated since 2006, the aim of the SUKIE-Study (Sukie = Säuglings- und Kinderernährung) is to give an update on the current breastfeeding rates in Austria and to update the national data for international comparison and use. This will provide evidence for further measures to promote breastfeeding.

## 2. Materials and Methods

### 2.1. Study Design

The study is a representative, longitudinal survey of breastfeeding and infant nutrition in Austria.

The survey was performed as an online questionnaire, and was also suitable for smartphones. The period for the infant nutrition survey was set to 12 months. At 4 time points—14 days, 4 months, 6 months and 12 months post-partum—participating mothers received a questionnaire. To derive the WHO “Infant and Young Child Feeding” indicators, the main questions were asked retrospectively at the respective time point. 

For this study, approval was obtained from the Ethics Committee of the Medical University of Vienna (Ref. 1303/2018) and all other relevant ethics committees in the Austrian provinces (Ref. 90/2018, Ref. 2358, Ref. 30-438ex17/18, Ref. GS1-EK-4/555-2018, Ref. EKB13-18, Ref. EKB13-18). The study is registered at Clinical-Trials.gov (Trial registration: NCT04137796. Registration date 24 October 2019). The recruitment and documentation of the participants was conducted analogously to the STROBE statement [21].

### 2.2. Subjects and Sample Selection

A total of 64 maternity wards (81% of 79 hospitals with delivery suites) across the whole country confirmed their voluntary participation. Some hospitals did not have the capacity (due to amalgamation of hospitals at this time) or had no interest in the study to support the recruitment period. Mothers were recruited mainly in puerperium in February/March 2019 in participating hospitals. Since no seasonal differences regarding breastfeeding are known, a representative sample during the recruitment period (February/March) was assumed. They were recruited and informed about the study by trained, multilingual (including Arabic and Bosnian-Croatian-Serbian) fieldworkers as well as by medical staff as approved by the ethical commission. Detailed study information and a registration tool were available online. To inform and facilitate participation of mothers who had given birth at home, the support of midwives was established. Mothers who had not given birth in one of the recruiting hospitals could also self-register via the study homepage if they were interested in participating. The study sample included mothers of legal age who gave birth during the recruitment period. Exclusion criteria for participation were mothers of new-borns in intensive care due to severe health problems. After an initial review of the inclusion and exclusion criteria, the mothers who had consented were asked to complete the 14-day questionnaire. Subsequently, another eligibility screening of the inclusion and exclusion criteria was conducted because more information was available from the participants after the first questionnaire (such as if in the meantime their babies have been admitted to intensive care).

### 2.3. Survey and Questionnaire Development

The survey evaluated the breastfeeding behavior and infant nutrition. Four questionnaires included questions about birth/puerperium; the infant (sex, height, weight); breastfeeding; exact age (in days and weeks) when water, tea, glucose water, infant formula and/or solid and semi-solid food was introduced; and the sociodemographic parameters and lifestyle of the mother. Most of the questions were closed ones (e.g., questions about the delivery process) with a few open-ended (e.g., questions about the age of the infant, height and weight). Information on measures such as the height and weight of the infant were self-measured or were obtained from the maternity card (Mutter-Kind-Pass).

Due to the importance of the international comparability of the data [14,21] the definitions of the WHO [22] were used. Individual questions were adapted to derive the “Infant and Young Child Feeding” (IYCF) indicators, provided by the WHO [22,23,24]. Questions and procedures from the German-speaking neighbor countries [19,20,25] were taken into account for a better comparability. The online format corresponded to current communication habits. Smartphone suitability of the questionnaires for mothers was very important to make data recording as simple as possible.

In order to test the user-friendliness of the questionnaire, a pilot study in two Austrian maternity wards (Graz and Vienna) were conducted over a period of one week. The comprehensibility of the main questions regarding WHO indicators was checked with a cognitive pretest (*n* = 15). In this pilot phase, the time to complete the survey (approximately 19 min) and the compliance within the target group were evaluated. Based on the feedback received during the pilot phase, the questionnaires were translated into Arabic and Turkish.

### 2.4. Outcomes

Primary outcomes of the study were the prevalence of breastfeeding after birth as well as the breastfeeding prevalence at 14 days, 4 months, 6 months and 12 months postpartum. Secondary outcomes were the duration of exclusive, predominant or partial breastfeeding, the assessment of infant feeding if the infant is not breastfed as well as the assessment of infant nutrition and complimentary feeding.

### 2.5. Indicators and Definitions Used in the Present Paper

The definitions and indicators used were based on the WHO indicators [22,23]. For this paper, data on breastfeeding were applied for 6 out of 15 WHO IYCF indicators, with a focus on breastfeeding in the first year of life [22,23]. Only indicator 11 was adapted for the age range (15 months instead of 23 months) due to our study population.

**Core WHO Indicators** [22,23]

- Indicator 1: Early initiation of breastfeeding describes the proportion of infants who were put within one hour of birth to the breast.
- Indicator 2: Exclusively breastfeeding <six months is defined as the proportion of infants 0–182 days of age who received exclusively breast milk (included expressed breast milk) and if necessary, additional oral rehydration solution, syrups (medicines, vitamins, minerals) or drops.

**Selected optional WHO Indicators** [22,23]

- Indicator 9: Children ever breastfed as a percentage of infants.
- Indicator 11: Age-appropriate breastfeeding is the proportion of infants 0–182 days of age who are exclusively breastfed and infants 183–438 days who received additionally to breast milk semi-solid, solid or soft foods.
- Indicator 12: Predominant breastfeeding <six months describes the proportion of infants 0–182 days of age who received the predominant source breastmilk but were allowed to receive other liquids such as water or water-based drinks.
- Indicator 13: Duration of breastfeeding as the median duration.
- Indicator 14: Bottle feeding describes the proportion of infants who were fed with a bottle in the first year of life.

In order to provide an accurate representation according to the WHO categories, the age (in days and weeks) of the infants when liquids other than breastmilk or complementary food were introduced was collected through detailed questions at the respective time point. For example: “How old was your baby when it received water/tea for the first time?”. The infants were categorized to the respective WHO definition. As soon as water, tea, infant formula, or other liquids were provided in addition to exclusive breastfeeding, the infants were classified into their respective higher categories (according to the following order: exclusive breastfeeding; breastfed and plain water/tea only; breastfed and formula; breastfed and complementary foods; and not receiving any breast milk).

In a further step, the Austrian recommendations on complementary feeding were taken into account for relevant indicators, as these deviate from the WHO recommendations. In Austria, the national recommendations for complementary feeding specify a possible introduction of solid food between the beginning of the fifth month and the end of the sixth month, depending on the child’s stage of development [26]. In order to consider the Austrian recommendations, an additional cut-off of 120 days for exclusive breastfeeding (Indicator 2), age-appropriate breastfeeding (Indicator 11), and predominant breastfeeding (Indicator 12) had to be set in the evaluation. 

### 2.6. Sample Size Calculation

The sample size was calculated considering a breastfeeding prevalence of 90% at baseline, as well as a 17% breastfeeding rate after one year, according to the data from 2006. In order to determine the breastfeeding prevalence of 17% after one year with an accuracy of ±2% (with 95% confidence interval (CI), 1600 mothers had to be recruited. The Austria-wide distribution of participating hospitals also ensured a regionally balanced composition of the sample.

### 2.7. Statistical Analysis

All statistical analyses were performed with R Statistical Software (version 4.0.2) [27]. A level of *p* < 0.05 was considered statistically significant. The data was analyzed by looking at categorical and metric variables. To describe categorical variables, frequencies or percentages were used, and for metric variables, medians and confidence interval or means (for age of introduction) were used. For inferential statistical evaluations, the chi-square test was applied. In order to account for differences between the samples and population compositions, the sample distribution with regard to education level and age was population-weighted using the R package “survey” [28]. This eliminates the bias regarding population statements and ensures that sample data are representative for the Austrian mother population. In the case of population-weighted analysis, the number (*n*) is not given in order to avoid the misleading impression that these are the direct sample results. 

The prevalence for the categorical WHO indicators was analyzed and the 95% CI was estimated. Data that were either fully available or could be comprehensibly completed were taken into account in the evaluation. In the case of missing data in connection with breastfeeding for single questionnaires, data were completed if this could be derived from the data of other time points of measurement. For statements on breastfeeding duration, underestimates were avoided due to the case of those mothers who were still breastfeeding at the end of data collection (right-censored data).

Since age was asked retrospectively, the individual time points of measurement were no longer considered in the analysis. Due to a possible second reminder, some questionnaires were completed later and the children were already older than one year.

## 3. Results

### 3.1. Sample Characteristics

**Figure 1:**
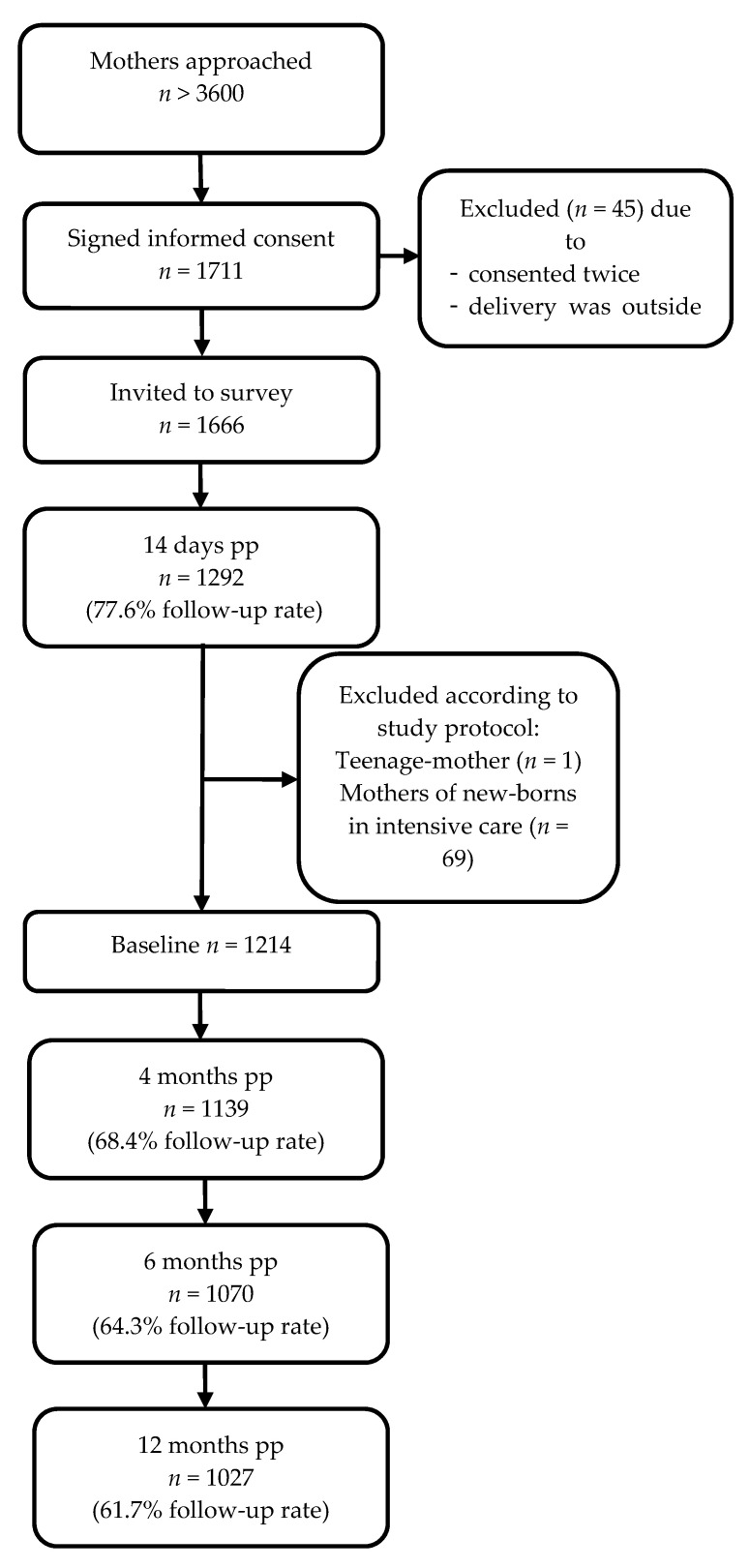
Flowchart of the sample selection process including follow-up rate of responding mothers (pp = post-partum).

After screening the inclusion and exclusion criteria according to the study protocol, a total of 1214 mothers were included in the analysis. This is less than the intended number, with the consequence of slightly larger confidence intervals. An overview of the participant flow is shown in Figure 1.

The median age of the mothers was 31 years (interquartile range (IQR) 28; 35). The youngest participant was 18 years old and the oldest was 48. One third of the mothers (30.0%) had a caesarean section. A minority (3.1%) were single mothers. A detailed distribution of age (in categories) and other characteristics of the study sample and the Austrian mothers’ population is shown in Table 1. The Austria-wide distribution showed a regionally balanced composition of the sample. The composition of the mothers’ sample with regard to the criteria of age (in categories) and education differed from the Austrian mother population according to STATISTIK AUSTRIA [29]. The population weighting ensures that no overrepresented sample group biased the results in overall statements for the maternal population. This also counteracts selection bias.

The majority of study participants gave birth in hospitals (98.4%). Only 1.6% (*n* = 19) delivered extramurally. Participating mothers who already had children (48.0%) had an average of two children (median = 2, min = 1, max = 7). Four out of five mothers (79.4%, *n* = 964) and 76.9% (*n* = 913) of fathers were born in Austria. Other countries of birth of the mother were mainly indicated as Germany (*n* = 49), Bosnia and Herzegovina (*n* = 22) and Hungary (*n* = 18). Only a few mothers used the translated questionnaires in Turkish (*n* = 4) and Arabic (*n* = 4).

### 3.2. Breastfeeding Prevalence in the First Year of Life

**Figure 2:**
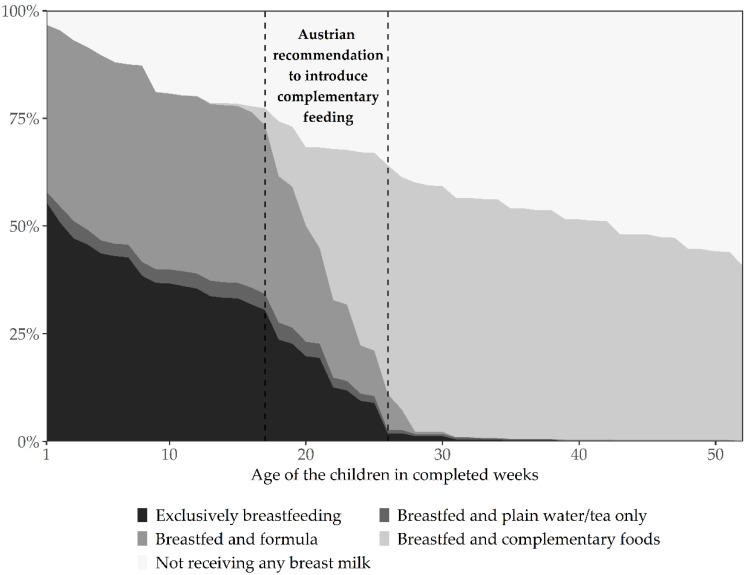
Breastfeeding practice by age of infants.

The results of the breastfeeding rates by breastfeeding practice in the first year of life are shown in Figure 2.

At one week of age, 96.7% (95% CI 95.6, 97.9) of infants were breastfed, at the age of four months (17 weeks), six months (26 weeks) and 12 months (52 weeks), 77.4% (95% CI 71.7, 83.0), 64.1% (95% CI 55.8, 72.5) and 40.8% (95% CI 31.8, 49.7) of infants were breastfed respectively. At the age of one week, 55.5% (95% CI 50.4, 60.6) of infants were exclusively breastfed. At 17 and 26 weeks of age, 30.5% (95% CI 28.0, 33.1) and 1.9% (95% CI 1.2, 2.5) respectively were exclusively breastfed. Figure 2 shows the decreasing proportion of “exclusively breastfeeding” in the first six months of life.

### 3.3. IYCF Indicators

The proportions of infants meeting the respective indicators are shown in Table 2. The infants included in this study were aged 0–15 months. For this reason, the indicators with an age range of 0–23 months were evaluated in relation to 0–15 months only. For three indicators (2, 11 and 12), a higher percentage of infants meeting the indicator criteria was observed when the Austrian recommendations on complementary feeding [27] were taken into account.

The data show that a large number of mothers (97.5%) had breastfed their child. However, an equally high percentage of infants (81.9%) were bottle-fed (Indicator 14). According to the responses of the mothers, 46.7% (95% CI 41.6, 51.8) of breastfed infants received infant formula, water, tea, or other liquids during the first days of life. These infants were not meeting the WHO definition of “exclusive breastfeeding”. Feeding formula was significantly more common (*p* < 0.001; χ^2^ = 15.6) in cesarean birth than in vaginal birth (68.4% vs. 42.3%). On average, infants received water or tea for the first time at the median age of 22 weeks (Table 3).

However, half of the infants (median) received infant formula for the first time in the first three days of life (IQR 2; 52).

The median duration of breastfeeding in Austria was 27 weeks (IQR 8; 52). However, this value refers only to the data of those mothers who ceased breastfeeding in the first year of life (right-censored data). At the end of the first year of life, 40.8% of infants were partially breastfed. Only two of these infants (0.17%) were still predominantly breastfed. The maximum duration of any breastfeeding was 61 weeks.

A large proportion of mothers (85.9%, 95% CI 84.4, 87.4) reported a skin-to-skin contact with their baby immediately after birth. Breastfeeding initiation within the first hour post-partum happened in 68.2% (95% CI 65.7, 70.7) of mothers.

## 4. Discussion

Breastfeeding is one of the most effective ways to protect the health of children and mothers and to promote healthy growth in early childhood [30]. This survey provides the first representative data for Austria in 15 years and an indispensable scientific basis for planning further measures to promote breastfeeding as well as for international comparison and use. The WHO European region has the lowest rate of breastfeeding worldwide [8,9]. The update of the breastfeeding data in Austria shows a high rate of breastfeeding initiation, but also a need for improvement in both total breastfeeding duration and exclusive breastfeeding.

### 4.1. Breastfeeding Prevalence in Comparisson with Previous Research

Breastfeeding initiation within the first hour of life is important for a newborn’s health and provides lifetime benefits [31]. The global breastfeeding collective states that globally only 43% of infants initiate breastfeeding within the first hour after birth. Austria has already almost reached the collective target for this rate of 70% [7].

Our data show a high rate of breastfeeding initiation at birth in Austria. At baseline the rate of any breastfeeding increased from 93.2% in the last study in 2006 [17], to 97.5% in this one. This initial breastfeeding rate is also high when compared with 11 European countries, where only Norway showed a higher rate (98%) [21]. The recently published German SuSe II-study reported an initial breastfeeding rate of 96.6% [19]. In both German-speaking countries, Germany and Switzerland, the breastfeeding prevalence dropped to about 41% towards the end of the first year of life. In order to reach the global target of continued breastfeeding for at least one year of 80% in 2030 [7], more efforts promoting breastfeeding are definitely needed. The data show that only two mothers were still predominantly breastfeeding one year after birth. This would indicate a need for a better education on the importance of introducing complementary feeding. In the present study, half of the infants received formula within the first three days of life. As shown by Hemmingway et al. [32], the combined feeding of breast milk and formula might be one of the reasons for early weaning. In our study, an early introduction of formula feeding was a common practice. This appears to impact on total breast feeding duration.

### 4.2. Exclusive Breastfeeding within the First six Months of Age

The results of the current study show that the 2025 Global Target for Nutrition for exclusive breastfeeding of at least 50% [33] is only achieved in the first two weeks of life. It is striking that in the current study, 46.7% of the infants received other liquids in addition to breast milk during the first days of life. Based on the WHO definition, these infants are not categorized as “exclusively breastfeeding” anymore [22].

In 2006, the proportion of exclusive breastfeeding at six months of age was 9.7% [17]. Previously, exclusive breastfeeding was recommended for six months [34]. The new data show that exclusive breastfeeding at six months was only 1.9%; however, it was still 9.0% after 5.5 months. This decrease can be explained by the fact that the Austrian guidelines for introducing complimentary food recommends that solid and semi-solid foods should not be given before the fifth month (completed 17th week), but also not after the end of the sixth month (26th week), depending on the child’s stage of development [26]. This recommendation is in line with the ESPGHAN (European Society for Paediatric Gastroenterology, Hepatology and Nutrition) recommendations [35].

Similar results were shown in Germany, where 8.3% of the infants at six months of age were exclusively breastfed [19]. Compared with other European countries, the rate of exclusively breastfed infants at six months is low in Austria. The prevalence of exclusive breastfeeding at six months of age in the Member States of the WHO European Region ranged between 0.7% (Greece) and 49.3% (Slovakia), based on data from 21 out of 53 countries from 1998 to 2013 [18]. A more recent review from 2019 with 11 European countries showed higher rates of exclusive breastfeeding at six months of age from 13% in Denmark to 39% in the Netherlands. Notably, Denmark used a different definition for full breastfeeding. The infant does still count as fully breastfed if it does not receive more than one formula feeding per week [21]. Based on WHO definitions, these infants would not be included in the exclusive breastfeeding category. This inconsistent use of definitions has already been addressed [18,21]. In high-income countries, the used indicators are rarely standardized and recall periods tend to be long [10]. This is the same in Austria, as the last country-wide survey was commissioned by the Ministry 15 years ago.

The attempt to compare data from different countries clearly shows the need of regular and standardized data collections using harmonized breastfeeding indicators [1,9,21]. Furthermore, only 36% of countries of the WHO regions have evaluated exclusive breastfeeding rates within the recommended time interval of five years [7]. Moreover, within the European countries there are huge differences between systematic monitoring of breastfeeding [21]. As already observed in previous studies, a clear distinction between exclusive, partial breastfeeding and all the other categories should be made [32,36]. Underestimation of the prevalence of supplementation with water, tea, and formula of breastfed infants can easily happen [36].

A methodologically consistent approach of data collection, taking into account WHO definitions, would be a major step towards the provision of comparable data. There should be a uniform, systematic, standardized monitoring of national breastfeeding rates, at least within European countries.

### 4.3. Strengths and Limitations of the Study

One of the major strengths of this study is the high rate of participation among both the clinics and the mothers all over the country. Some hospitals were not interested in participating in the study without giving reasons. Most of the study participants gave birth in the hospital. In addition, we were able to reach those who gave birth outside the hospital. This proportion is identical to the Austrian population, where 98.4% of children were born in hospital in 2019 [29]. We recorded a high response rate and a good distribution of participants from the federal states in Austria at baseline. This was facilitated by a sensitive approach when making first contacts with the mothers. The design of the study also took into account the multiple daily challenges of mothers by providing a smartphone solution to complete the questionnaire.

In questionnaire surveys, selection bias and social desirability bias cannot be completely excluded. Due to frequent emotional discussions on breastfeeding, a selection bias may occur, as the study could primarily address women that are already interested in breastfeeding. With the intention of preventing this possible selection bias, study staff were trained in communicating with the target group in general terms of “infant and child nutrition” instead of focusing on the topic of breastfeeding. In order to avoid social desirability bias in the indicator questions, some variables (breastfeeding categories, e.g., exclusively breastfeeding) were not asked directly but indirectly via several detailed questions. This facilitated a more precise allocation to the breastfeeding categories.

Another unique strength of this study is the categorization of infant age by weeks when liquids and food were introduced. Thus, we can accurately determine the breastfeeding prevalence as well as the respective breastfeeding category at each week within the infants’ first year of life.

Nevertheless, some potential limitations have to be mentioned. In this study a higher proportion of mothers with a higher educational background took part, similar to other studies in the health environment. The underrepresentation of members of the lower educated group was taken into consideration when analyzing the data to ensure representativeness. Possible language barriers were addressed in advance by multilingual field workers and the translations of information materials and questionnaires. However, the offer of non-German questionnaires was used only to a small extent.

One of the major challenges was the requirement of having to file for ethics approval in every federal state. This needs to be taken into consideration when planning further research in this field.

## 5. Conclusions

This study investigates a sensitive period within the first 1000 days of life. The current results provide an indispensable national update on the current breastfeeding rate. Austria’s high initial breastfeeding rate decreased during the first year of life, as was also seen in other countries. Overall breastfeeding duration, as well as exclusive breastfeeding rates, still need improvement. Standardized data collection with harmonized breastfeeding indicators should continue to be conducted regularly in the future. Further analysis about factors influencing breastfeeding are necessary. They will provide better insights into the possible entry points to support and promote breastfeeding.

## Figures and Tables

**Table 1:** Characteristics of the Austrian mothers’ population [29] and study sample (*n* = 1214) at different time points.

Characteristic of Mothers	Population in Austria (%)	*n*	Sample 14 days (%)	*n*	Sample 4 mos. (%)	*n*	Sample 6 mos. (%)	*n*	Sample 12 mos. (%)	*n*
**Age at delivery (year)**										
18–24	10.8 *	9171	7.7	93	7.0	72	6.0	58	5.0	47
25–29	29.0	24,621	26.8	325	25.8	265	26.0	253	26.1	246
30–34	34.8	29,550	38.9	472	39.9	409	40.4	394	40.6	383
35–39	19.7	16,770	21.1	256	21.8	224	21.9	213	22.6	213
≥40	4.4	3731	5.5	68	5.5	56	5.7	56	5.7	54
**Educational level**										
Low (ISCED 0–2)	10.1 **	8229	4.0	49	2.6	27	2.0	19	2.2	21
Middle (ISCED 3–4)	47.1 **	38,474	52.0	631	51.7	530	50.7	494	49.5	467
High (ISCED 5–8)	13.1 **	10,722	44.0	534	45.7	469	47.3	461	48.3	455
**State/Residence**										
Vienna	23.5	19,935	23.3	283	19.8	203	19.4	189	18.7	176
Vorarlberg	5.1	4319	4.7	57	4.7	48	4.7	46	5.0	47
Tyrol	8.9	7522	7.9	96	8.0	82	8.4	82	8.4	79
Styria	12.9	10,970	14.7	179	14.9	153	15.0	146	14.9	141
Salzburg	6.8	5780	7.3	89	7.5	77	7.5	73	7.4	70
Upper Austria	17.7	15,057	16.1	196	17.1	175	17.2	167	17.7	167
Lower Austria	17.2	14,652	17.8	216	19.3	198	19.7	192	19.5	184
Carintia	5.3	4485	5.0	61	5.8	60	5.3	52	5.6	53
Burgenland	2.6	2232	3.0	37	2.9	30	2.8	27	2.8	26

* 20–24 year olds; ** data from 2014; mos. = months; ISCED = International Standard Classification of Education (UNESCO).

**Table 2:** IYCF indicators for breastfeeding according to WHO [22,23] and Austrian recommendations [26] in %.

	According to WHO Definitions *		According to Austrian Recommendations **	
	%	95% CI	%	95% CI
Early initiation of breastfeeding (<1 h) (Ind. 1)	68.2	66.1; 71.3	68.2	66.1; 71.3
Exclusive breastfeeding (Ind. 2)	1.9	1.2; 2.5	30.5	28.2; 33.4
Children ever breastfed (Ind. 9)	97.5	96.4; 98.5	97.5	96.4; 98.5
Age-appropriate breastfeeding (Ind. 11)	1.7	1.1; 2.4	29.4	26.8; 32.1
Predominant breastfeeding (Ind. 12)	1.8	1.2; 2.5	3.7	2.6; 4.9
Bottle feeding (Ind. 14)	81.9	79.5; 84.2	81.9	79.5; 84.2

* Indicator 2 and 12: under six months (<183 days); ** Indicator 2 and 12: under four months (<120 days) = Austrian guidelines of complementary feeding; Ind. = Indicator.; WHO = World Health Organization; CI = confidence interval

**Table 3:** Age in completed weeks when other liquids/foods were introduced.

Age in Weeks	Min	Q1	Median	Mean	Q3	Max
Introduction infant formula	0	0	0	7	7	55
Introduction water and/or tea	0	2	22	22	26	60
Introduction complementary food	9	17	22	21	24	56

Q1 = first quartile; Q3 = third quartile

## Data Availability

The data presented in this study are available on request from the corresponding author.
